# Effect of 9,12-Octadecadiynoic Acid on Neurobehavioral Development in *Caenorhabditis elegans*

**DOI:** 10.3390/ijms22168917

**Published:** 2021-08-18

**Authors:** Tun-Chieh Chen, How-Ran Chao, Ching-Ying Wu, Yun-Ru Lai, Chu-Huang Chen, Tohru Yoshioka, Wen-Li Hsu, Ming-Hsien Tsai

**Affiliations:** 1 Department of Internal Medicine, Kaohsiung Municipal Ta-Tung Hospital, Kaohsiung Medical University, No. 68, Jhonghua 3rd Rd, Cianjin District, Kaohsiung 80145, Taiwan; idchentc@gap.kmu.edu.tw; 2 Division of Infectious Diseases, Department of Internal Medicine, Kaohsiung Medical University Hospital, Kaohsiung Medical University, No. 100, Shih-Chuan 1st Road, Kaohsiung 80708, Taiwan; 3 School of Medicine, College of Medicine, Kaohsiung Medical University, No. 100, Shih-Chuan 1st Road, Kaohsiung 80708, Taiwan; 4 Department of Environmental Science and Engineering, College of Engineering, National Pingtung University of Science and Technology, No.1, Shuefu Road, Pingtung 91201, Taiwan; hrchao@mail.npust.edu.tw; 5 Emerging Compounds Research Center, General Research Service Center, National Pingtung University of Science and Technology, No.1, Shuefu Road, Pingtung 91201, Taiwan; 6 Institute of Food Safety Management, College of Agriculture, National Pingtung University of Science and Technology, No.1, Shuefu Road, Pingtung 91201, Taiwan; 7 Department of Dermatology, Kaohsiung Municipal Ta-Tung Hospital, Kaohsiung Medical University Hospital, Kaohsiung Medical University, No. 68, Jhonghua 3rd Rd, Cianjin District, Kaohsiung 80145, Taiwan; fu.0888@gmail.com; 8 Department of Cosmetic Science, Chang Gung University of Science and Technology, No 261, Wenhua 1st Rd, Taoyuan 33303, Taiwan; 9 Regenerative Medicine and Cell Therapy Research Center, Kaohsiung Medical University, No. 100, Shih-Chuan 1st Road, Kaohsiung 80708, Taiwan; chromics.yunru@gmail.com (Y.-R.L.); yoshioka@kmu.edu.tw (T.Y.); 10 Vascular and Medicinal Research, Texas Heart Institute, 6770 Bertner Avenue, Houston, TX 77030, USA; cchen@texasheart.org; 11 New York Heart Research Foundation, 200 Old Country Road, Mineola, NY 11501, USA; 12 Institute for Biomedical Sciences, Shinshu University, 3-1-1 Asahi Matsumoto, Nagano 390-8621, Japan; 13 Graduate Institute of Medicine, College of Medicine, Kaohsiung Medical University, No. 100, Shih-Chuan 1st Road, Kaohsiung 80708, Taiwan; 14 Department of Child Care, College of Humanities and Social Sciences, National Pingtung University of Science and Technology, No.1, Shuefu Road, Pingtung 91201, Taiwan

**Keywords:** human breast milk, lipidomics, 9,12-octadecadiynoic acid, neurodevelopment, adaptive behavior, *Caenorhabditis elegans*

## Abstract

Human breast milk lipids have major beneficial effects: they promote infant early brain development, growth and health. To identify the relationship between human breast milk lipids and infant neurodevelopment, multivariate analyses that combined lipidomics and psychological Bayley-III scales evaluation were utilized. We identified that 9,12-octadecadiynoic acid has a significantly positive correlation with infant adaptive behavioral development, which is a crucial neurodevelopment to manage risk from environmental stress. To further clarify the biological function of 9,12-octadecadiynoic acid in regulating neurodevelopment, *Caenorhabditis elegans* (*C. elegans*) was used as a model to investigate the effect of 9,12-octadecadiynoic acid on neurobehavioral development. Supplementation with 9,12-octadecadiynoic acid from the L1 to L4 stage in larvae affected locomotive behaviors and foraging ability that were not socially interactive, implying that 9,12-octadecadiynoic acid is involved in regulating the serotonergic neuronal ability. We found that supplementary 0.1 μM 9,12-octadecadiynoic acid accelerated the locomotive ability and foraging ability via increasing the expression of serotonin transporter *mod-1.* Antioxidant defense genes, *sod-1*, *sod-3* and *cyp-35A2* are involved in 9,12-octadecadiynoic acid-induced motor neuronal activity. Nevertheless, supplementary 9,12-octadecadiynoic acid at concentrations above 1 μM significantly attenuated locomotive behaviors, foraging ability, serotonin synthesis, serotonin-related gene expressions and stress-related gene expression, resulting in the decreased longevity of worms in the experiment. In conclusion, our study demonstrates the biological function of 9,12-octadecadiynoic acid in governing adaptive behavioral development.

## 1. Introduction

The World Health Organization (WHO) suggests a global strategy for infant and young child feeding, and that breastfeeding is helpful to improve infant neurodevelopment, growth and health. Human breast milk contains a mixture of substances that contribute to its nutritional value, and human breast milk lipids supply the fundamental source of energy and necessary nutrients for infants [1]. Human breast milk lipids are mainly composed of triglycerides, which are esters derived from three fatty acids and glycerol. In a small population, polyunsaturated fatty acids (PUFAs) constitute only 0.8% to 26% of triglycerides in human breast milk; they are considered some of the most important lipids required for infant short-term and long-term neurodevelopmental outcomes [2]. Although we are continuously learning more about the role of lipids, especially PUFAs in infant neurodevelopment, lipids represent a large group of macronutrients constituting a majority of components that remain largely unstudied and poorly understood.

**Figure 1:**
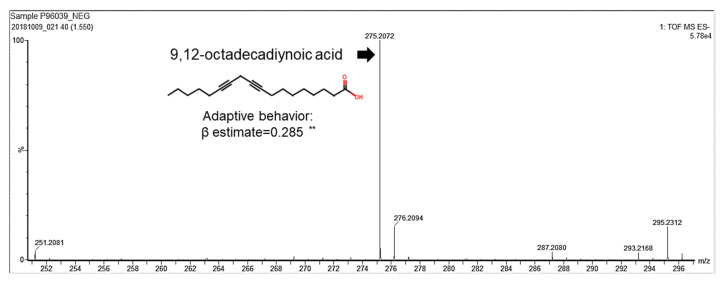
9,12-octadecadiynoic acid reveals a positive correlation with adaptive behavior. Multivariate analyses between 9,12-octadecadiynoic acid and adaptive behavior of the Bayley-III developmental score adjusted for mother’s age, body mass index and parity number of birth (*n* = 100). Spearman correlation coefficient of 9,12-octadecadiynoic acid vs. adaptive behavior, correlation coefficient, *β* estimate = 0.28875; ** *p* < 0.01.

We identified a correlation between human breast milk lipid composition and infant neurodevelopment. Milk samples were collected from the study subjects within the first month after giving birth in the Kaoping area in Taiwan. Lipids were further isolated from human breast milk and analyzed for their components. Infants’ and children’s neurodevelopmental assessment scores were measured according to Bayley-III scales. The relationship between human breast milk lipid components and neurodevelopment were analyzed by multiple regression. Notably, 9,12-octadecadiynoic acid (C_18_H_28_O_2_) had a significantly positive correlation coefficient (*β* estimate = 0.28875) with an adaptive behavioral development (Table 1) and was identified from thousands of human breast milk lipids (Figure 1).

9,12-Octadecadiynoic acid can be a derivative from 9,12-octadecadienoic acid (linoleic acid), revealing triple bonds at positions 9 and 12 of linoleic acid. So far, the biological function of 9,12-octadecadiynoic acid is still unclear. This study utilized *Caenorhabditis elegans* (*C. elegans*) as a model to investigate how 9,12-octadecadiynoic acid affected neurobehavioral development. Consequently, we wanted to investigate the effect of the amount of 9,12-octadecadiynoic acid the infant receives from breastfeeding; therefore, worms were supplemented with 9,12-octadecadiynoic acid only from the L1 to L4 stage. Our results demonstrate in part the effect of 9,12-octadecadiynoic acid on biological function.

## 2. Results

### 2.1. Effects of 9,12-Octadecadiynoic Acid Supplementation on Neurobehavioral Indicators in Worms

Our study found that 9,12-octadecadiynoic acid was positively correlated with adaptive behavioral development, which is a complex behavioral development in response to environmental demands. Adaptive behavioral development is defined as the collection of conceptual, social and practical skills learned by people to enable them to function in their everyday lives [3], implicating impact on children’s adaptive capacity to manage risks in unsafe environments. In *C. elegans*, adaptive behavioral development is an important response to either environmental or internal physiological changes [4]. Neuronal activity has been shown to interact with environmental adaptability that extends further to adaptive behavioral development [5]. Therefore, neurobehavioral indicators are crucial to reflect the condition of the neural system, and the effect of 9,12-octadecadiynoic acid on neurobehavioral development in *C. elegans* was further investigated.

**Figure 2:**
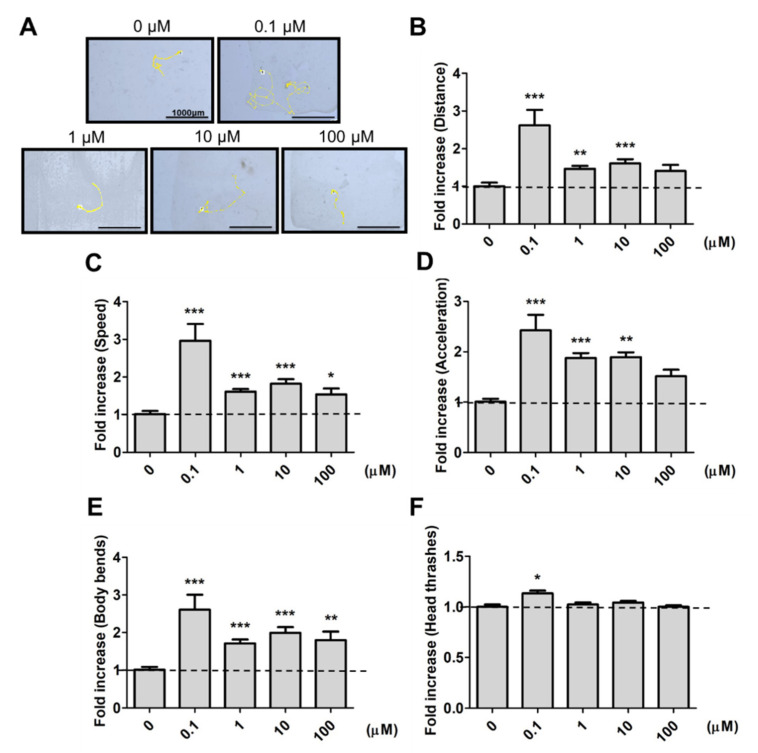
Locomotion evaluation after larval intake of 9,12-octadecadiynoic acid. (**A**) Worm tracking, (**B**) moving distance, (**C**) moving speed, (**D**) acceleration, (**E**) body bends and (**F**)head thrashes. Data (*n* = 30) were presented as the fold value compared to the control group (mean ± SEM, * p < 0.05; ** p < 0.01; *** p < 0.001).

To avoid the possible metabolic effects on 9,12-octadecadiynoic acid, we firstly examined the worm’s feeding protocol in Supplementary Figure S1, and confirmed the growth of worms from the L1 to L4 stage with the feeding of UV-killed *E. coli OP50*. To identify whether 9,12-octadecadiynoic acid affected neuronal activity in *C. elegans*, locomotive behaviors were evaluated by worm tracking, distance moved, body bends, head thrashes, moving speed and acceleration after larval intake of 9,12-octadecadiynoic acid until young L4 stage. As shown in Figure 2, supplementation with 9,12-octadecadiynoic acid using a dose of 0.1 μM significantly enhanced worms’ locomotive behaviors, but supplementing with 9,12-octadecadiynoic acid doses above 1 μM resulted in the significant inhibition of their locomotive behaviors. Interestingly, the spatial pattern of worm tracking with 0.1 μM 9,12-octadecadiynoic acid supplementation revealed more complications than with other concentrations (Figure 2A). 

In addition, an adaptive response to environmental changes can be reflected in foraging activity [6]. We further detected the effect of 9,12-octadecadiynoic acid on foraging ability. A similar result was also observed for foraging behavior; supplementary 9,12-octadecadiynoic acid promoted foraging activity at 0.1 μM, but this activity was delayed at concentrations above 1 μM (Supplementary Figure S2). Our results imply that a low concentration supplementation with 9,12-octadecadiynoic acid in *C. elegans* potentially accelerated motor neuronal activity due to the enhancement of neurobehavioral indicators in worms.

### 2.2. Effects of 9,12-Octadecadiynoic Acid Supplementation on Aggregative Behavior in Worms

**Figure 3:**
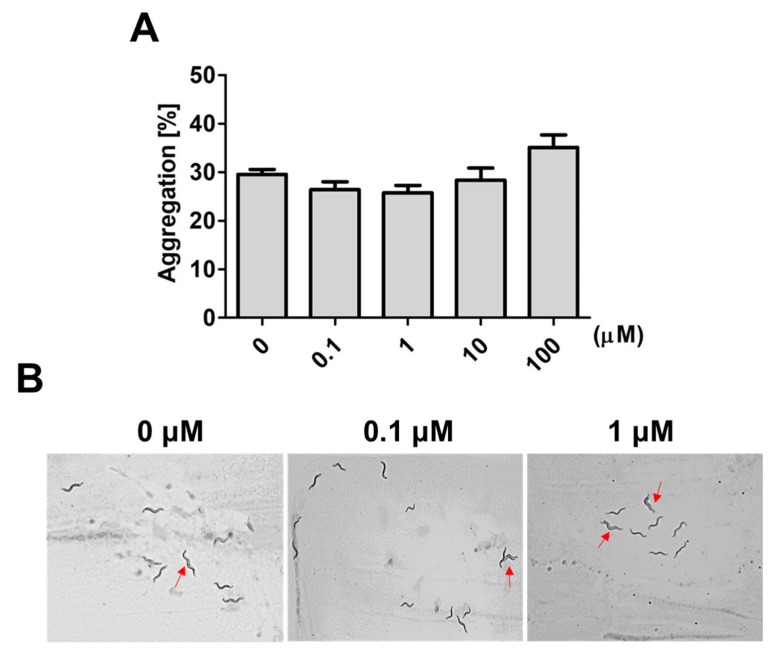
Aggregation behavior analysis after larval intake of 9,12-octadecadiynoic acid. (**A**) Aggregation behavior of the worms exposed to different concentrations of 9,12-octadecadiynoic acid pretreatment. The data represent the average of three independent experiments (mean ± SEM). (**B**) Aggregation of the worms (red arrow) on plates.

In worms, social interactions, such as aggregative behavior, can be regulated by sensory neurons that respond to environmental stress and maintain survival [7,8]. A family of small molecules, the ascarosides, play important roles as chemical signals regulating aggregative behavior in worms. Therefore, we further investigated whether 9,12-octadecadiynoic acid affected aggregative behaviors that are regulated by ascarosides in *C. elegans*. After larval intake of 9,12-octadecadiynoic acid from the L1 to L4 stage, aggregative behavior in 20 worms was measured. Although we observed that the aggregation number of worms was increased at high concentrations of supplementary 9,12-octadecadiynoic acid, there was no statistically significant difference with 9,12-octadecadiynoic acid supplementation (Figure 3A,B). It could be that sensory neurons are not importantly involved in 9,12-octadecadiynoic acid-regulated neurobehavioral development. Our results supported that supplementation with 9,12-octadecadiynoic acid under 100 μM in *C. elegans* did not significantly affect aggregative behavior.

### 2.3. Supplementary 9,12-Octadecadiynoic Acid in Worms Influences Serotonin Synthesis and Serotonin-Related Gene Expression

There are four major neurons in *C. elegans* larvae, a pair of pharyngeal neurosecretory motor neurons, MSNs, and a pair of chemosensory neurons, ADFs [9]. According to our findings, supplementation with 0.1 μM 9,12-octadecadiynoic acid in worms promoted locomotive behaviors. Motor neuronal activity, especially NSM, could be regulated by supplementation with 9,12-octadecadiynoic acid. Serotonin (5-hydroxytryptamine, 5-HT), which is synthesized in NSMs, belongs to a neurotransmitter group responsible for regulating food sensory activity in a new environment [10]. Therefore, we supposed that serotonin synthesis or serotonin-related gene expression in worms may be affected by supplementing with 9,12-octadecadiynoic acid.

**Figure 4:**
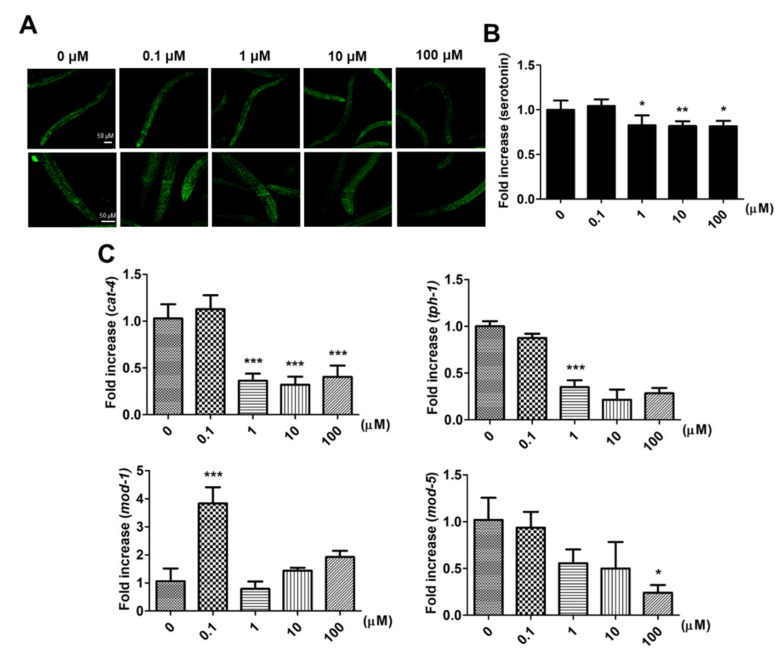
Effect of Effects of 9,12-octadecadiynoic acid on serotonin synthesis and serotonin-related gene expression in *C. elegans*. (**A**) Analysis of serotonin (green) in *C. elegans* by immunohistochemistry staining. The intensity of emitted fluorescence was quantified by using an Olympus confocal microscope as the (**B**) (mean ± SD, * p < 0.05; ** p < 0.01). (**C**) Integrated gene serotonin-related expression profiles tested after larval intake of 9,12-octadecadiynoic acid. Values of serotonin-related gene expression were normalized using actin mRNA and represent means relative to the control (mean ± SD, * p < 0.05; *** p < 0.001).

After larval intake of 9,12-octadecadiynoic acid from the L1 to L4 stage, the distribution of serotonin is shown in Figure 4A, and the fluorescence signals of serotonin were especially revealing in the pharyngeal area where MSNs are located. The quantification of the serotonin fluorescence signals revealed the following: Although supplementary 0.1 μM 9,12-octadecadiynoic acid enhanced locomotive behaviors, serotonin synthesis was not increased (Figure 4B). We further detected the expression of tryptophan hydroxylases, *cat-4* and *tph-1*, the key enzymes for serotonin biosynthesis [11], and found that supplementation with 0.1 μM 9,12-octadecadiynoic acid did not accelerate the levels of *cat-4* and *tph-1* (Figure 4C).

Serotonin transporters, MOD-1 and MOD-5, facilitate the influence of locomotive behaviors in *C. elegans* via modulating serotonin dynamics [12,13]. To investigate how supplementary 0.1 μM 9,12-octadecadiynoic acid affected locomotive behaviors, the expressions of *mod-1* and *mod-5* were also examined. Our results indicated that only the level of *mod-1* was significantly enhanced by supplementation with 0.1 μM 9,12-octadecadiynoic acid (Figure 4C). However, supplementation with 9,12-octadecadiynoic acid above 1 μM significantly decreased serotonin synthesis and serotonin-related gene expression (Figure 4B,C). These results were similar to the effect of 9,12-octadecadiynoic acid on locomotive behaviors and suggested that the neurosecretory motor neuronal activity in worms is modulated by 9,12-octadecadiynoic acid-regulated serotonin dynamics. This finding implies that 9,12-octadecadiynoic acid significantly regulates locomotive behaviors, especially MSN. Therefore, MOD-1, a serotonin transporters, is involved in 0.1 μM 9,12-octadecadiynoic acid-increased motor neuronal activity and alters neural plasticity in locomotive behaviors. 

### 2.4. Supplementary 9,12-Octadecadiynoic Acid in Worms Influences Stress-Related Gene Expression

**Figure 5:**
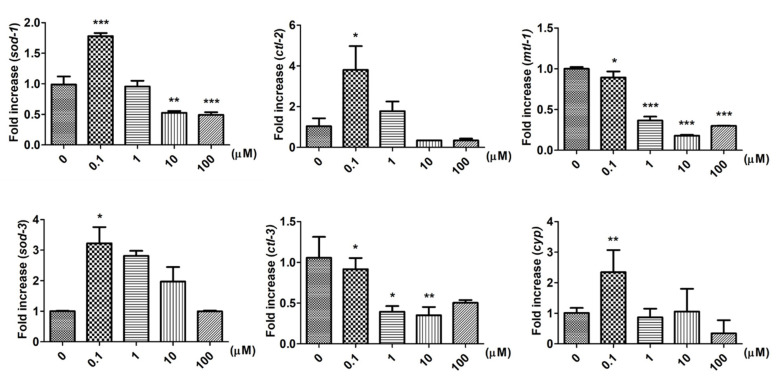
Effect of 9,12-octadecadiynoic acid on stress-related genes in *C. elegans*. Integrated gene stress-related expression profiles tested after larval intake of 9,12-octadecadiynoic acid. Values of stress-related gene expression were normalized using actin mRNA and represent means relative to the control (mean ± SD, * p < 0.05; ** p < 0.01; *** p < 0.001).

The adaptive behavior of biological systems is especially evident in response to environmental stress [14]. To maintain survival during times of environmental stress, stress-related genes, such as superoxide dismutases (SODs), catalases (CATs) or xenobiotic metabolism enzymes, which are important and indispensable in the entire defense strategy of antioxidants [11], are involved in regulating adaptive responses to a changing environment [15,16,17]. In addition, those stress-related genes are also closely associated with the regulation of motor neuronal activity [18,19]. We next identified whether stress-related genes were involved in 9,12-octadecadiynoic acid-regulated adaptive behavioral development. Similarly, our findings revealed that the levels of *sod-1*, *sod-3*, *ctl-2* and *cyp-35A2* (*cyp)* were significantly increased by supplementation with 0.1 μM 9,12-octadecadiynoic acid, but decreased by supplementary 9,12-octadecadiynoic acid above 1 μM in contrast to the control (Figure 5). The expressions of the following genes were attenuated in the presence of supplementary 9,12-octadecadiynoic acid, including *ctl-3* and *mtl-1* (Figure 5). We further confirmed the expression of *sod-1*, *sod-3*, *ctl-2* and *cyp-35A2* in middle adulthood (10th day) of worms which were supplemented with 0.1 μM 9,12-octadecadiynoic acid during the larval stage. A mere *sod-1*, *sod-3* and *cyp-35A2*, the levels of those genes were significantly accelerated in middle adulthood (Supplementary Figure S3).

The activation of multiple stress pathways is regulated by FOXO transcription factor *daf-16*, whose known targets include *cyp-34A9*, *sod-3* and *mtl-1* [20]; we additionally identified the expression of *daf-16*. Despite no effect of treatment with 0.1 μM 9,12-octadecadiynoic acid on *daf-16* expression, supplementation at concentrations above 1 μM significantly restrained the expression of *daf-16* (Supplementary Figure S4). The decreased level of *daf-16* potentiated the downregulation of stress-related genes with high doses of 9,12-octadecadiynoic acid supplementation. As a result, stress-related genes are involved in 9,12-octadecadiynoic acid-regulated adaptive behavioral development.

### 2.5. Effects of 9,12-Octadecadiynoic Acid Supplementation on Lifespan in Worms

**Figure 6:**
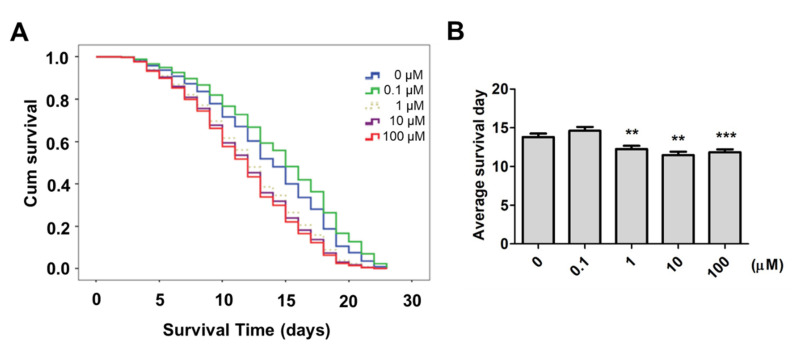
Lifespan evaluation after larval intake of 9,12-octadecadiynoic acid. Worms were treated at different concentrations from 0 μM to 100 μM of 9,12-octadecadiynoic acid in the larval stage. (**A**) Kaplan–Meier curves with univariate analyses for worms with treatment by different concentrations of 9,12-octadecadiynoic acid (*n* = 120). (**B**) Average survival days of three independent experiments were analyzed by (**A**). The data were presented as compared to the control group (mean ± SD, ** p < 0.01; *** p < 0.001).

Childhood experiences and the consequences on neurobiological, psychosocial and somatic conditions have effects across the entire lifespan [21]. Our study suggests that nematodes exposed to supplementary 9,12-octadecadiynoic acid had lasting impacts on locomotive behaviors, serotonin synthesis, serotonin-related gene expression and stress-related gene expression. We further evaluated whether nematodes that were supplemented with 9,12-octadecadiynoic acid from the L1 to L4 stage displayed altered lifespans. Our results showed that there was no significant increase in lifespans and average survival days in worms with supplementary 0.1 μM 9,12-octadecadiynoic acid (Figure 6). Nevertheless, supplementary 9,12-octadecadiynoic acid at concentrations above 1 μM resulted in significantly shorter lifespans and average survival days than was the case for the control (Figure 6). According to our results, worms that were supplemented by 9,12-octadecadiynoic acid from the L1 to L4 stage experienced an impact on their longevity.

## 3. Discussion

Our study firstly demonstrated the biological function of 9,12-octadecadiynoic acid which has been identified from human breast milk in regulating neurobehavioral development, and the effects of 9,12-octadecadiynoic acid on the larval stage of *C. elegans* across their lifespan. Based on the analysis of lipidomics and multiple regression, 9,12-octadecadiynoic acid of human breast milk significantly correlated with adaptive behavioral development that displayed a positive effect at a low dose. *C. elegans was* chosen as the experimental organism in our study because it is a readily available animal model, it has a precise neural system and a well-defined distribution of neurons. We mimicked infant obtainment of 9,12-octadecadiynoic acid from breastfeeding, and *C. elegans* larvae were supplemented by different concentrations of 9,12-octadecadiynoic acid from the L1 to L4 stage. Adaptive behavioral development in worms was evaluated by locomotive behaviors, foraging ability, aggregative behavior and chemotaxis. Despite no statistically significant effect on aggregative behavior, our findings pointed out that supplementation with a low dose of 0.1 μM 9,12-octadecadiynoic acid upregulated locomotive ability and foraging ability via the government of motor neuronal activity.

Serotonin transporter *mod-1* and stress-related genes *sod-1*, *sod-3* and *cyp-35A2* were involved in 9,12-octadecadiynoic acid-induced motor neuronal activity. Supplementation with 0.1 μM 9,12-octadecadiynoic acid during the larval stage also significantly increased levels of *sod-1*, *sod-3* and *cyp-35A2* in middle adulthood (Supplementary Figure S3), but there was no significant effect on the worms’ lifespan. This implies that the roles of *sod-1*, *sod-3* and *cyp-35A2* have specific roles in regulating motor neuronal activity with a supplementary low dose 9,12-octadecadiynoic acid. However, supplementary 9,12-octadecadiynoic acid at concentrations above 1 μM significantly downregulated locomotive behaviors, foraging ability, serotonin synthesis, serotonin-related gene expression and stress-related gene expression, leading to the decreased longevity of worms in the experiment. Previous studies indicated that *daf-16* is involved in regulating the lifespan in *C. elegans* [22]; deficient *daf-16* restrains the lifespan of the worms [23]. According to our study, supplementary 9,12-octadecadiynoic acid at concentrations above 1 μM led to significantly shorter lifespans and average survival days than was the case for the control. As shown in Figure 5 and Supplementary Figure S4, *daf-16* and its regulated antioxidant genes, such as *sod-3* and *mtl-1,* were downregulated by supplementation above 1 μM 9,12-octadecadiynoic acid. Therefore, 9,12-octadecadiynoic acid’s influence on the lifespan of *C. elegans* may be due to the expression of *daf-16*.

Although we provided supplementary 9,12-octadecadiynoic acid, the effect on lifespan, reactive oxygen and nitrogen species (RONS) production was not observed at the L4 stage. Based on the oxidative stress theory of aging, RONS production that cannot be quenched by antioxidant defenses contributes to age-associated functional losses and even death [24]. Supplementation above 1 μM 9,12-octadecadiynoic acid in worms restrained antioxidant genes, *sod-1*, *sod-3*, *ctl-2*, *ctl-3, mtl-1, cyp-35A2* and *daf-16* and RONS production may be not quenched following the induction of the aging process. Interestingly, supplementation with 0.1 μM 9,12-octadecadiynoic acid promoted locomotive behavior, foraging ability, neurotransmitter serotonin dynamics and antioxidant gene’ activity, but the lifespan was increased without significance. Previous studies have illustrated that childhood experiences have consequences on neurobiological, psychosocial and somatic conditions that are evident across the lifespan [21]. It seems that adverse childhood experiences have more profound influence than favorable childhood experiences; thus, providing a larger long term impact on lifespan. Indeed, adverse childhood experiences such as sexual and physical abuse or neglect constitute a massive stressor with long-lasting adverse effects on the brain, mental and physical health [21].

The concentration of 9,12-octadecadiynoic acid in human breast milk could not be measured exactly, because the content of fatty acids in human breast milk is dependent on the intake from the mother. If 9,12-octadecadiynoic acid can be derived from linoleic acid sources, then vegetable oils such as corn, sunflower, soybean and peanut oils, which supply linoleic acid for the production of human breast milk, then these are suggested sources of intake for breastfeeding mothers. However, the dose of these vegetable oils for breastfeeding needs to be further investigated, because excess intake in childhood potentially decreases neural activity and attenuates lifespan according to our study. Additionally, supplementary low dose 9,12-octadecadiynoic acid by tablets, capsules or drinks may be used to supply an exact dose for lactating mothers. We will further define the efficient dose of 9,12-octadecadiynoic acid in mammals in future studies.

Dissimilar to genetics, proteomics or metabolomics which have been established for years, lipidomics is now gaining popularity as the novel technology of mass spectrometry becomes more and more prevalent. Although we reviewed a huge range of lipids in our experiments, many of the biological functions of those lipids are still unclear. Actually, lipid derivatives, or their isomers, may have biological functions completely different from the original lipid. Thus, our study clarified the biological function of 9,12-octadecadiynoic acid in governing neurodevelopment, and provided information in support of the Sustainable Development Goals (SDGs) for the issue of children’s health care.

## 4. Material and Methods

### 4.1. Lipid Analysis and Neurodevelopmental Test

A total of 100 mother-infant pairs were included in our study on the basis of exclusive or partial breastfeeding, and human breast milk samples were collected from the subjects within the first month after giving birth in the Kaoping area in Taiwan. Lipids were further isolated from human breast milk and analyzed for their components utilizing liquid chromatography combined with quadrupole-time of flight mass spectrometer (LC/MS/MS, ESI-qTOF, Waters, Milford, CT, USA). To specifically perform analysis of fatty acids, electrospray ionization recognized lipids with negative (ES- model) charge. Furthermore, the Bayley Scales of Infant and Toddler Development, Third Edition (Bayley-III) was a tool for measuring the infant neurodevelopment of children at the age of 10 months. The Bayley-III score has five domains, including cognitive, language, and motor scales that are assessed by the child’s psychometrics [25]. To further investigate the associations of fatty acids and the five domains of Bayley-III scores, we analyzed descriptive statistics and Spearman correlation and performed univariate and multivariate analyses, which were described in Section 4.9. Statistical Analysis.

The adaptive behavior scales were assessed using two-parent report questionnaires that were answered by the parents according to the child’s psychometrics, including the attainment of practical skills necessary for a child to function independently and meet environmental demands [26]. 

### 4.2. C. elegans Culture and 9,12-Octadecadiynoic Acid Feeding Protocol

*C. elegans* (Bristol strain N2) was attained from the Caenorhabditis Genetics Center (CGC), cultivated on nematode growth medium (NGM) agar plates, and fed with UV-killed *Escherichia coli OP50* to prevent the potential confounding effects of bacterial metabolism. *C. elegans* was dissolved in alkaline bleach solution to collect eggs and hatched on the NGM plates with food at 20 °C for 48 h to obtain age-synchronized populations.

To mimic infant receipt of 9,12-octadecadiynoic acid from breastfeeding, synchronized L1 larvae were seeded onto NGM plates and fed with UV-killed *E. coli* supplementing different concentrations of 9,12-octadecadiynoic acid until young L4 stage as previously described [11]. Five nominal 9,12-octadecadiynoic acid concentrations, which were referred to in previous studies [27], were utilized in this study, namely, 0 μM (control), 0.1 μM, 1 μM, 10 μM and 100 μM. After larval intake of 9,12-octadecadiynoic acid, the nematodes were prepared for further experiments.

### 4.3. Evaluation of Locomotive Behaviors

After supplementing with 9,12-octadecadiynoic acid from L1 to L4 stage, the locomotive behaviors of the worms were identified by tracking, distance, head thrashes, body bends, moving speed and acceleration. All locomotion experiments were detected using an NIS-Elements AR Object Tracking module (NIKON, Tokyo, Japan).

### 4.4. Foraging Behavior

After supplementing with 9,12-octadecadiynoic acid from L1 to L4 stage, the worms were transferred to chemical-free 9 cm plates for the foraging behavior assay as previously described [11]. Before the assay, a UV-killed E. coli lawn was applied circularly within a 0.5 cm radius from the center. The worms were evenly placed 4 cm from the center. Each test plate was loaded with 12 worms and five replicates were created for each treatment. After 2, 4, 6 and 24 h of incubation at 20 °C, the number of worms that had touched to the E. coli colony was calculated in each plate utilizing an NIS-Elements dissecting microscope (NIKON, Tokyo, Japan). The attainment level of worms was acquired by dividing the number of worms that touched the food source by the total number of worms on the plate.

### 4.5. Evaluation of Aggregation

NGM plates were seeded with an overnight culture of UV-killed *E. coli OP50* and allowed to dry for 2 days at room temperature as a bacterial lawn for aggregation assays. After larval intake of 9,12-octadecadiynoic acid until young L4 stage, we placed 20 worms onto the lawn and left them at 20 °C for 3 h. Aggregation behavior was quantified as the number of animals that were in touch with two or more animals at >50% for 3 s of their body length [7].

### 4.6. Immunohistochemistry Staining

Worms were fixed by incubating with 10% formaldehyde (Sigma-Aldrich, St. Louis, MO, USA) for 30 min and permeabilized by 0.25% Triton-X100 treatment. The fixed worms were then briefly washed with PBS and incubated overnight at 4 °C in PBS containing 5% BSA with serotonin antibody (Novus Biologicals, Centennial, CO, USA). The fixed worms were subsequently incubated with secondary antibody for 1 h and then mounted on coverslips that were then inverted and fixed onto glass slides by utilizing antifade mounting reagent (Biotium, Fremont, CA, USA). Fluorescence imaging was detected by a confocal microscope (FV1000, Olympus, Tokyo, Japan).

### 4.7. Gene Expression Assay

Total RNA was extracted from *C. elegans* with the Trizol reagent (Invitrogen, Waltham, MA, USA). Reverse transcriptase reactions required 1 μg RNA to synthesize complementary cDNA utilizing a Reverse Transcription kit (Invitrogen, USA) according to the manufacturer’s protocol. Incubation conditions included 10 min at 25 °C, 120 min at 37 °C and 5 min at 85 °C. The resulting cDNAs were used to identify the level of serotonin-related gene expression (*mod-1*, *mod-5*, *cat-4*, *tph-1*) and stress-related gene expression (*sod-1*, *sod-3*, *ctl-2*, *ctl-3*, *mtl-1*, *cyp*) by quantitative PCR using SybrGreen PCR Master Mix Kit (Applied Biosystems, Waltham, MA, USA) and specific primers as previously described [11]. 

### 4.8. Lifespan Assay

The tested worms were moved to new 60 mm agar dishes, which were seeded with *E. coli OP50*, and were marked as day 0 when growing to the L4 stage. Moreover, the worms were transferred to new agar dishes daily during the first 4–5 days to avoid mixing between generations. The worms that showed no response to gentle prodding with a platinum wire and no pharyngeal pumping were considered dead. Three biological replicates were performed and a total of 120 worms were assayed [28].

### 4.9. Statistical Analysis

The correlation between lipid components and infant adaptive behavioral development was utilized as the independent variable in further multivariate analyses with a generalized estimating equation (GEE) model in which we adjusted for confounding factors such as mother’s age, mother’s highest level of education, family income, mother’s smoking behavior, pre-pregnancy BMI, parity number of birth, period of time living in Kaoping area, newborn’s sex, gestational age (weeks), birth weight, birth length and head circumference. The beta (β) estimates, 95% confidence intervals and *p*-values were calculated using Statistical Analysis System (SAS version 9.4, Cary, NC, USA). GraphPad Prism was utilized to create bar charts; error bars pointed out standard deviations unless otherwise noted. Analysis of variance and Student’s t-tests were used to compare the differences between groups. In addition, survivorship analysis was performed using the Statistical Product and Service Solutions (SPSS, version 12.0, Chicago, IL, USA). A *p*-value of less than 0.05 for the difference between groups was considered statistically significant.

## 5. Conclusions

Despite many studies that have indicated that human breast milk lipids have positive effects on infant neurodevelopment, growth and health, our study is the first to define the mechanism of human breast milk lipid, 9,12-octadecadiynoic acid, in modulating neurodevelopment. The utilization of multivariate analyses combined lipidomics and psychology, and identified that 9,12-octadecadiynoic acid has a significantly positive correlation with infant adaptive behavioral development, which is a crucial neurodevelopment to manage the risk from environmental stress. To investigate the effect that the amount of 9,12-octadecadiynoic acid the infant receives from breastfeeding, worms were supplemented with different concentrations of 9,12-octadecadiynoic acid from L1 to L4 stage. Our study demonstrated that 9,12-octadecadiynoic acid at low doses under 0.1 μM potentiates enhancement of serotonergic neurons and antioxidant defenses, improving adaptive responses to a changing environment. In contrast, supplementation above 1 μM 9,12-octadecadiynoic acid in worms may decrease adaptive responses, significantly attenuating serotonergic neuronal activity, antioxidant defense and lifespan. In conclusion, our study demonstrated the biological function of 9,12-octadecadiynoic acid and its potential effect on infant adaptive behavioral development.

## Figures and Tables

**Table 1:** Multivariate analyses between lipid components and the adaptive behavior of the Bayley-III developmental score (*n* = 100) adjusted for mother’s age, body mass index and parity number of birth.

Description	Compounds Description (rt_*m/z*)	Formula	Category	β estimate (*p*-Value)
Bullatacinone	1.05_621.4340	C_37_H_66_O_7_	Fatty acid	0.247 (*0.013*)
9,12-octadecadiynoic acid	1.55_275.2061	C_18_H_28_O_2_	Fatty acid	0.285 (*0.005*)
16:1(5Z)	2.02_253.2221	C_16_H_30_O_2_	Fatty acid	−0.203 (*0.037*)

## Data Availability

All relevant data are within the manuscript.

## Supplementary Materials

The following are available online at https://www.mdpi.com/article/10.3390/ijms22168917/s1.
