# Supplementary material for: Effect of 9,12-Octadecadiynoic Acid on Neurobehavioral Development in Caenorhabditis elegans
Source: Int J Mol Sci. 2021 Aug 18;22(16):8917. doi: 10.3390/ijms22168917 (PMC8396327; doi:10.3390/ijms22168917)
Supplement: Supplementary file 1 [file ijms-22-08917-s001.zip › ijms-1227412-supplementary.pdf]

Supplementary Materials to:

Effect of 9,12-octadecadiynoic acid on neurobehavioral development in *Caenorhabditis elegans*

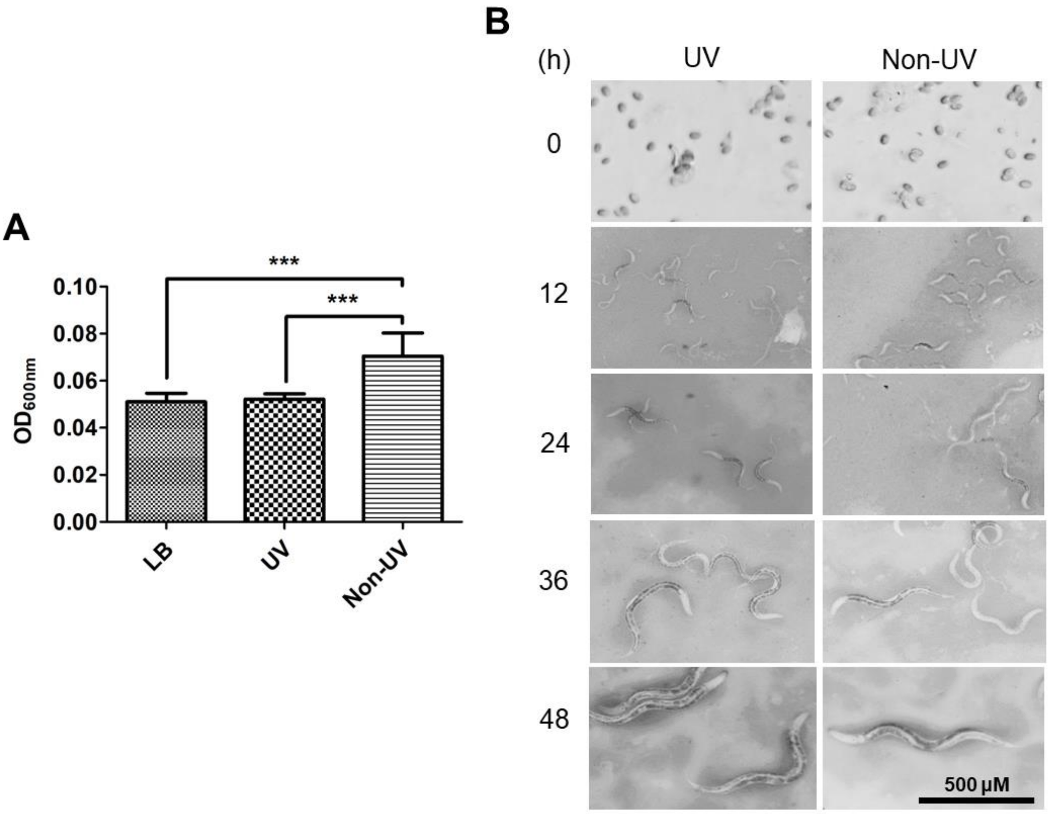

Supplemental Figure S1. *E. coli OP50* with or without UV treatment doesn't affect the growth of L1 larva. NGM plates were seeded with an overnight culture of *E. coli OP50* as a bacterial lawn. After treating with or without UV overnight, small pieces of bacterial lawn (5 mm^3) were cut from NGM plates, and then cultured in LB medium for 3 h at 37 °C. (A) The growth of bacteria in liquid culture was measured by the optical density at 600 nm (OD_{600nm}). The growth of *E. coli OP50* was significantly restrained by UV treatment. (mean ± SD, ****p < 0.001*) (B) *C. elegans* eggs were seeded onto NGM plates containing *E. coli OP50* and UV-killed *E. coli OP50* respectively. The growth of worms were observed at the indicated time points.

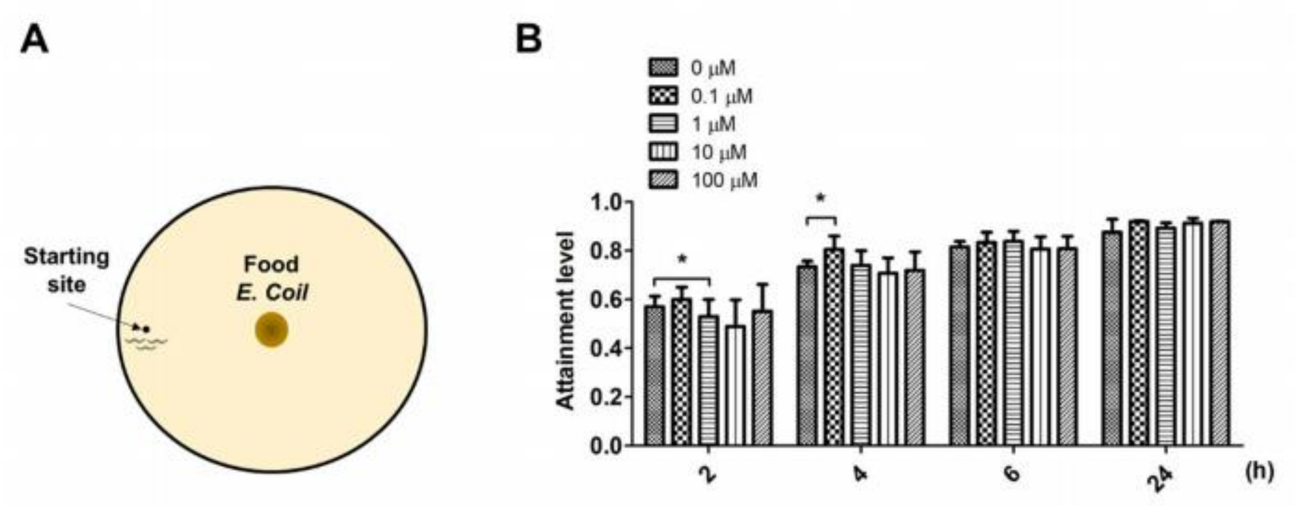

Supplemental Figure S2. Effect of 9,12-octadecadiynoic acid on foraging behavior in *C. elegans.* (A) The methodological design of foraging behavior. (B) Influence of 9,12-octadecadiynoic acid in attainment level of worms (mean ± SEM, *p < 0.05).

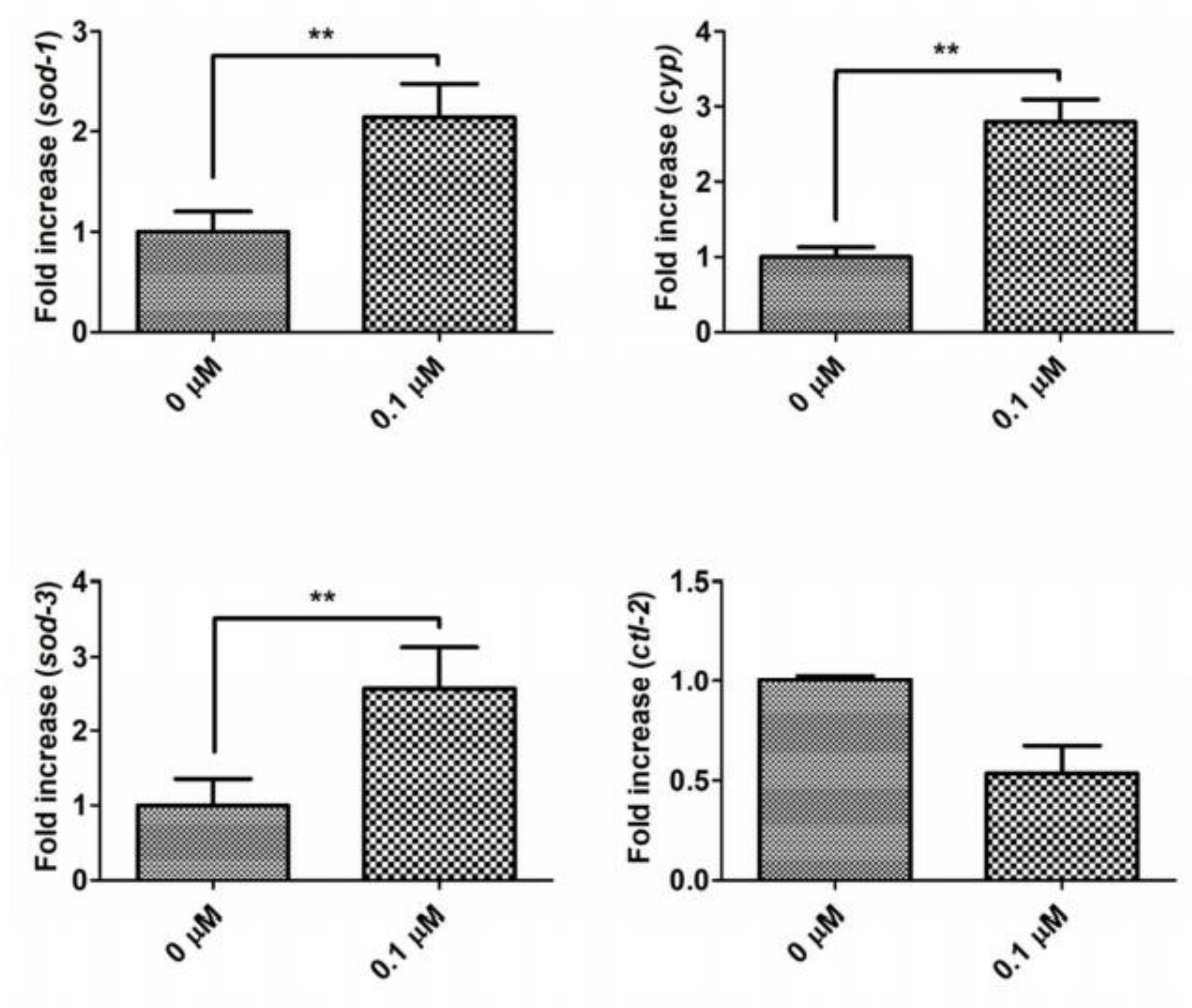

Supplemental Figure S3. Analysis of the expression of *sod-1*, *sod-3*, *ctl-2* and *cyp-35A2* in middle adulthood of worms. Worms are supplemented by 0.1 μM of 9,12-octadecadiynoic acid from L1 to L4 stage, and analyzed the expression of *sod-1*, *sod-3*, *ctl-2* and *cyp-35A2* at 10th day of adults (mean ± SD, $^{**}p < 0.01$).

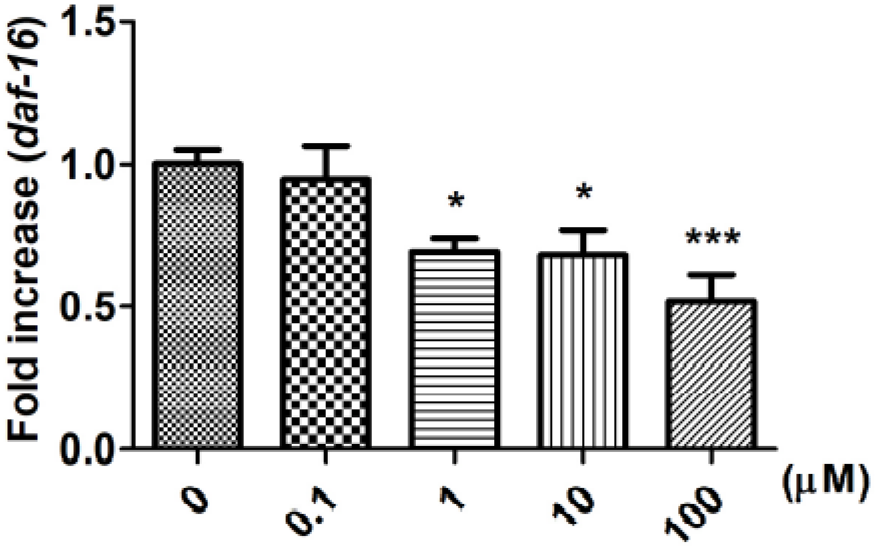

Supplemental Figure S4. Analysis of the expression of *daf-16* after larval intake of 9,12-octadecadiynoic acid (mean ± SD, **p* < 0.05; ****p* < *0.001*).
